# High Carriage of *tetA*, *sul1*, *sul2* and *bla*_TEM_ Resistance Genes among the Multidrug-resistant Uropathogenic *Escherichia coli* (UPEC) Strains from Malaysian Patients

**DOI:** 10.21315/tlsr2024.35.2.10

**Published:** 2024-07-31

**Authors:** Jia-Jin Chin, Hui-Mei Lee, Shuet-Yi Lee, Yin-Ying Lee, Choy-Hoong Chew

**Affiliations:** Department of Allied Health Sciences, Faculty of Science, Universiti Tunku Abdul Rahman (UTAR), 31900 Kampar, Perak, Malaysia

**Keywords:** *bla*_TEM_, Multidrug Resistance, *sul1*, *sul2*, *tetA*, *bla*_TEM_, Perintang Pelbagai Dadah, *sul1*, *sul2*, *tetA*

## Abstract

The rapid emergence of multidrug-resistant (MDR) uropathogenic *Escherichia coli* (UPEC) strains pose a critical challenge in urinary tract infection (UTI) treatments. However, little work elucidated the resistance mechanisms of the MDR UPEC clinical strains in Malaysia. Therefore, this study aimed to determine the antimicrobial susceptibility profiles and the prevalence of antimicrobial resistance genes among the UPEC strains. Polymerase chain reactions were conducted to detect the presence of 6 antimicrobial resistance genes among 60 UPEC strains. Meanwhile, the antimicrobial resistance profiles against 9 antimicrobials were examined through the Kirby-Bauer disk diffusion method. In this study, the MDR isolates accounted for 40.0% (24/60), with the highest prevalence of resistance towards ampicillin (43/60; 71.7%), followed by tetracycline (31/60; 51.7%), nalidixic acid (30/60; 50.0%), co-trimoxazole (20/60, 33.3%), ciprofloxacin (19/60, 31.7%), levofloxacin (16/60, 21.6%) and chloramphenicol (10/60, 16.7%). In contrast, low resistance rates were observed among minocycline (1/60; 1.7%) and imipenem (0/60; 0.0%). *bla*_TEM_ was the most prevalent gene (36/60; 60.0%), followed by *tetA* (27/60; 45.0%), *sul2* (25/60; 41.7%), *sul1* (13/60; 21.7%) and *tetB* (8/60; 13.3%). Surprisingly, *bla*_SHV_ was not detected among the UPEC isolates. The MDR, ampicillin and tetracycline-resistant isolates were significantly associated with a higher prevalence of *tetA*, *sul1*, *sul2* and *bla*_TEM_. In contrast, *tetB* displayed no significant relationship with any of the antimicrobials tested. The patient’s age and gender were not the risk factors for the carriage of the resistance genes. Our findings identified the common resistance genes carried by the antimicrobial resistant UPEC isolates and provide valuable insights into developing the best antibiotic prescription regime to treat UTIs in our local scene.

## Abstrak

Kemunculan pesat strain *Escherichia coli* (UPEC) uropathogenik tahan *multidrug* (MDR) menimbulkan cabaran kritikal dalam rawatan jangkitan saluran kencing (UTI). Di Malaysia, kami menghadapi kekurangan informasi penyelidikan tentang mekanisme rintangan strain klinikal MDR UPEC. Oleh itu, kajian ini bertujuan untuk menentukan profil kerentanan antimikrob dan kelaziman gen rintangan antimikrob dalam kalangan strain UPEC di Malaysia. Tindak balas rantai polimerase telah dijalankan untuk mengesan kehadiran 6 gen rintangan antimikrob di antara 60 strain UPEC. Sementara itu, profil kerentanan antimikrob terhadap 9 antimikrob telah diperiksa melalui kaedah Kirby-Bauer. Dalam kajian ini, isolat MDR menyumbang 40.0% (24/60), dengan prevalens rintangan tertinggi terhadap ampicillin (43/60; 71.7%), diikuti oleh tetrasiklin (31/60; 51.7%) dan asid nalidiksik (30/60; 50.0%). Sebaliknya, kadar kerentanan yang tinggi diperhatikan di kalangan minocycline (59/60; 98.3%) dan imipenem (60/60; 100.0%). *bla*_TEM_ adalah gen yang paling lazim (36/60; 60.0%), diikuti oleh *tetA* (27/60; 45.0%), *sul2* (25/60; 41.7%), *sul1* (13/60; 21.7%) dan *tetB* (8/60; 13.3%). Yang menghairankan, *bla*SHV tidak dikesan di kalangan isolat UPEC. MDR, ampicillin dan tetracycline dikaitkan dengan prevalens *tetA, sul1, sul2* dan *bla*_TEM_ yang tinggi. Sebaliknya, *tetB* tidak menunjukkan hubungan yang signifikan dengan mana-mana antimikrobial yang diuji. Umur dan jantina pesakit bukanlah faktor risiko untuk membawa gen rintangan. Penemuan kami mengenal pasti gen rintangan biasa yang dibawa oleh pengasingan UPEC yang tahan antimikrob dan memberikan pandangan berharga untuk membangunkan rejim preskripsi antibiotik terbaik untuk merawat UTI di tempat kejadian tempatan kami.

**Highlights**

- Multidrug-resistant (MDR) uropathogenic *Escherichia coli* (UPEC) accounts for 40.0% of the isolates obtained, with the highest prevalence of resistance towards ampicillin, followed by tetracycline and nalidixic acid.
- *bla*_TEM_ is the most prevalent antibiotic resistance gene detected, followed by *tetA* and *sul2*. However, *bla*_SHV_ is not present among the UPEC isolates.
- The MDR, ampicillin and tetracycline-resistant isolates is significantly associated with a higher prevalence of *tetA, sul1, sul2* and *bla*_TEM_. The patient’s age and gender are not risk factors for the carriage of the resistance genes.

## INTRODUCTION

Urinary tract infection (UTI) represents one of the most frequently encountered microbial infections in humans that is predominantly caused by uropathogenic *Escherichia coli* (UPEC) (Maniam *et al*. 2022; Yang *et al*. 2022). While antimicrobial therapy remains the mainstay of treating and ameliorating the clinical symptoms of UTIs, the rise of multidrug resistance (MDR) among the UPEC strains makes UTI treatments progressively more challenging and expensive (Halaji *et al*. 2022; Maniam *et al*. 2022). The emergence of MDR UPEC strains is assumed to be driven by the widespread of antimicrobial resistance genes through mobile genetic elements such as transposons, integrons and conjugative plasmids (Rozwadowski & Gawel 2022).

In recent years, the UPEC strains have gained greater resistance towards firstline antimicrobials. For instance, trimethoprim-sulfamethoxazole, which serves as the mainstay for uncomplicated cystitis treatment, is less effective in countries including Pakistan (82%), Mexico (72.7%) and Mongolia (70.9%) due to the high resistance rates (Ramírez-Castillo *et al*. 2018; Kot 2019). Furthermore, ciprofloxacin, the empirical oral prescription for uncomplicated pyelonephritis, also shows a profound level of resistance in Ethiopia (85.5%), Taiwan (79.5%) and Thailand (65.4%) (Kot 2019; Tewawong *et al*. 2020; Lin *et al*. 2021). High resistance to amoxicillin-clavulanic acid, which is recommended for treating mild and moderate pyelonephritis, has been reported in countries such as Jordan (83%), Turkey (50.9%) and France (37.6%) (Lavigne *et al*. 2016; Kot 2019; Yılmaz & Aslantaş 2020). While the resistance rates of the UPEC strains are relatively lower for trimethoprim-sulfamethoxazole (34.1%), ciprofloxacin (27.0%) and amoxicillin-clavulanic acid (13.4%) in Malaysia, the resistance rates of amoxicillin-clavulanic acid and ciprofloxacin have increased from 13.2% and 26.3% in 2019 to 13.4% and 27.0% in 2020, respectively (Institute for Medical Research 2020).

Production of beta-lactamase is a widely known resistance mechanism of gram-negative bacteria, including UPEC strains (Xiao *et al*. 2019; Zhu *et al*. 2022). Beta-lactamase is a hydrolytic enzyme that cleaves the amide bond of the four-membered ring structure of beta-lactams antibiotics such as penicillins, cephalosporins, carbapenems and monobactams (Bush & Bradford 2020; Ibrahim *et al*. 2021). Common beta-lactamases include SHV-(sulfhydryl reagent variable) and TEMbeta-lactamases, which are encoded by the *bla*_SHV_ and *bla*_TEM_ genes (Gundran *et al*. 2019). TEM was first identified in *E. coli* isolated from a patient named Temoniera (Mansouri & Ramazanzadeh 2009). To date, hundreds of their variants are identified and often associated with isolates co-resistant to other classes of antibiotics, such as cotrimoxazole and fluoroquinolones (Bush & Bradford 2020; Castanheira *et al*. 2021; Salah *et al*. 2019).

Among the non-beta lactam antimicrobials, a high prevalence of tetracycline and sulphonamides such as cotrimoxazole are commonly observed among the UPEC strains (Bunduki *et al*. 2021; Mortazavi-Tabatabaei *et al*. 2019). The rising of tetracycline resistance is often associated with the acquisition of *tetA* and *tetB* efflux genes, which encode for membrane-associated proteins to export tetracycline from the cells (Chopra & Roberts 2001). On the other hand, sulphonamide resistance is typically driven by the acquisition of dihydropteroate synthase (DHPS) enzymes that are encoded by *sul* genes (Xu *et al*. 2020). Among the four plasmid-borne *sul* genes, *sul1* and *sul2* were more widely disseminated in geographical areas such as Europe, Canada, Iran, Poland and China than *sul3* and *sul4* (Adamus-Białek *et al*. 2018; Arabi *et al*. 2015; Blahna *et al*. 2006; Xu *et al*. 2020). These *sul* genes are co-located with other resistance genes (e.g., *tetA* and *bla*_TEM_ genes) on the same plasmids, suggesting that these plasmids may also aid in co-selecting other resistance genes (Poirel *et al*. 2018).

Surveillance of the antimicrobial resistance profiles and the resistance genes is crucial in combating the spreading of these antimicrobial resistant UPEC isolates. While the Malaysian National Surveillance of Antibiotic Resistance (NSAR) programmes have been established since 2000 to monitor the resistance profiles of the UPEC strains, little research revealed the prevalence of resistance genes in relation to the host factors such as age and gender (Ministry of Health Malaysia 2017). Therefore, this study is aimed to investigate the prevalence of *the resistance genes (bla*_TEM_, *bla_SHV_, tet and sul)*, and to determine the antibiotic resistance profile as well as the association between the phenotypic and genotypic data among the UPEC isolates in Malaysia. Here, we highlight the host age and gender differences in association with the antimicrobial gene profiles of the UPEC strains collected from Malaysian patients.

## MATERIALS AND METHODS

### Sample Collection

This research study was approved by the Ministry of Health Malaysia, with the reference number KKM/NIHSEC/P21-31(4). A total of 60 UPEC isolates were randomly collected from the patients’ urine specimens in Raja Permaisuri Bainun Hospital in 2020. All isolated bacterial had significant bacteriuria of more than 100,000 colony-forming units/mL and underwent bacterial identification through the Microflex^®^ LT/SH MALDI-TOF biotyper (Bruker, Germany).

### Antimicrobial Susceptibility Testing

The antimicrobial susceptibility profiles of UPEC isolates against ampicillin (10 μg), tetracycline (30 μg), minocycline (30 μg), nalidixic acid (30 μg), ciprofloxacin (5 μg), levofloxacin (5 μg), co-trimoxazole (25 μg), chloramphenicol (30 μg) and imipenem (10 μg) were conducted via the Kirby-Bauer disk diffusion method. The phenotypic profiles of the isolates were determined as described by the Clinical Laboratory Standards Institute (CLSI) guideline 2021. For analysis, isolates that showed intermediate resistance were also treated as resistant. The MDR isolates were defined as the UPEC isolates that showed resistance to 1 or more antimicrobial agents in 3 or more different antimicrobial categories (Magiorakos *et al*. 2012).

### Genomic DNA Extraction

All the template deoxyribonucleic acids (DNAs) were extracted through the fast-boil method, as described by (Kor *et al*. 2013). All extracted DNA samples had an A260/A280 ratio between 1.8 to 2.0 when measuring using NanoDrop™ 1000 Spectrophotometer (Thermo Scientific, United States).

### Detection of Antimicrobial Resistance Genes

A total of six antimicrobial resistance genes, including *bla*_SHV_, *bla*_TEM_, *tetA*, *tetB*, *sul1* and *sul2* were examined through 3 duplex polymerase chain reaction (PCR) assays (Table 1). The PCR assays were carried out in a total volume of 25 μL containing a final concentration of 1X buffer, 1.25 mM to 1.5 mM of magnesium chloride (MgCl_2_), 0.1 mM to 0.2 mM of each deoxynucleotide triphosphates (dNTPs), 1.25 U of *Taq* polymerase, 180 ng of template DNA and 0.5 μM primers (except for *tetA and tetB* primers; 0.3 μM and *bla*_TEM_ primers; 1 μM). All primer sequences and PCR conditions were illustrated in Table 1. The PCR products were resolved on 1.5% (w/v) agarose gel prestained with EtB“Out” nucleic acid staining solution at 90 volts for approximately 45 min. The gel images were then visualised and captured using the ChemiDoc™ XRS+ with Image Lab™ software (Bio-Rad, United States).

### Statistical Analysis

All statistical analyses were computed and analysed using the Statistical Package for the Social Sciences (SPSS) version 26 statistical software (IBM, United States). Pearson’s Chi-square test or Fisher’s exact test was conducted to analyse the categorical variables. A *p-*value < 0.05 was considered statistically significant throughout this study.

## RESULTS

### Demographic Profiles of the Study Population

Among the 60 UPEC isolates, 73.3% (44/60) were collected from female patients, whereas 26.7% (16/60) were collected from male patients (Table 2). For analysis, the host age was divided into three age groups: 14 years old and below, 15–59 years old and 60 years old and above. Most of the isolates were collected from the age group 60 years old and above (31/60; 51.7%), followed by the age group 15–59 years old (25/60; 41.7%) and 14 years old and below (4/60; 6.7%) (Table 2).

### Antimicrobial Resistance Profiles

Out of the nine antimicrobials tested, the highest resistance rate was observed among ampicillin (43/60; 71.7%), followed by tetracycline (31/60; 51.7%) and nalidixic acid (30/60; 50.0%) as shown in Table 3. The UPEC isolates also displayed greater resistance towards co-trimoxazole (20/60; 33.3%), ciprofloxacin (19/60; 31.7%), levofloxacin (16/60; 26.7%), chloramphenicol (10/60; 16.7%), but to a lesser extent towards minocycline (1/60; 1.7%). All the UPEC isolates were susceptible to imipenem. Alarmingly, 40.0% (24/60) of the isolates were MDR (Table 3).

### Prevalence of Antimicrobial Resistance Genes

PCR amplification of the resistance genes revealed that most of the UPEC isolates exhibited *bla*_TEM_ (36/60; 60.0%), but none of them contained *bla*_SHV_ (Table 4). Over 40% of the isolates conferred *tetA* (27/60; 45.0%) and *sul2* (25/60; 41.7%), but less than 20% of them carried *sul1* (13/60; 21.7%) and *tetB* (8/60; 13.3%).

### Association between Antimicrobial Resistance Phenotypes and Resistance Genes

Table 4 shows that the ampicillin and tetracycline-resistant isolates harboured a higher prevalence of *tetA*, *sul1*, *sul2* and *bla*_TEM_. On the other hand, *tetA*, *sul1* and *sul2* were more frequently detected among the nalidixic acid and co-trimoxazole-resistant isolates. Meanwhile, *sul1* and *bla*_TEM_ were more commonly found among the ciprofloxacin and levofloxacin-resistant isolates (Table 4). On the contrary, *tetB* displayed no significant relationship with any of the antimicrobials tested. Overall, the MDR isolates carried more *tetA* (19/24; 79.2%), *sul1* (12/24; 50.0%), *sul2* (16/24; 66.7%) and *bla*_TEM_ (19/24; 79.2%) (all *p <* 0.05) as shown in Table 4.

### Association between Antimicrobial Resistance Genes and Host Factors

The UPEC isolates collected from female patients carried a higher prevalence of *tetA* (20/44; 45.5%), *tetB* (7/44; 15.9%) and *bla*_TEM_ (28/44; 63.6%) (Table 5). In contrast, higher occurrences of *sul1* (6/16; 37.5%) and *sul2* (7/16; 43.8%) were observed among the male patients. However, Pearson’s Chi-square test demonstrated no significant relationship (*p >* 0.005) between the resistance genes and host gender (Table 5).

Table 5 shows that half of the resistance genes tested were prevalently found among the age group 15–59, including *tetA* (13/25; 52.0%), *sul1* (7/25; 28.0%) and *bla*_TEM_ (16/25; 64.0%). Meanwhile, *sul2* (2/4; 50.0%) and *tetB* (6/31; 19.4%) were more frequently detected among the age group 14 years and below and 60 years and above, respectively. No resistance gene was significantly correlated with host age (Table 5).

## DISCUSSION

Antimicrobial resistance crisis represents one of the primary health threats in Malaysia due to the indiscriminate use of antimicrobials (Haque *et al*. 2022; Naeemmudeen *et al*. 2021). Out of the nine antimicrobials tested, the UPEC strains displayed the highest prevalence of resistance towards ampicillin (43/60; 71.7%) (Table 3). This finding was consistent with the recent systematic review conducted by Naeemmudeen *et al*. (2021), in which the resistance rates of the ampicillin ranged from 68.0% to 100.0%. Ampicillin has been widely prescribed to treat *E. coli* infection in humans worldwide (Chen *et al*. 2019), which may explain its high resistance rate in the present study. Among the three quinolones antimicrobials, 50.0% of the isolates were resistant nalidixic acid (first-generation quinolone), followed by ciprofloxacin (second-generation quinolone) and levofloxacin (third-generation quinolone) (Table 3). Our results conform with the fact that the newer generations of quinolones have higher potency and a larger spectrum of activities than the older generations (Millanao *et al*. 2021; Suaifan *et al*. 2022). Furthermore, the high imipenem susceptibility rate (100.0%) observed in the current study was in accordance with the previous findings (Lin *et al*. 2021; Yılmaz & Aslantaş 2020). Carbapenems are typically served as the last-resort antibiotics to treat severe cases of UTI (Rozwadowski & Gawel 2022).

The prevalence of MDR isolates in this study (24/60; 40.0%) was higher than those reported in Libya (33.2%) and Turkey (34.6%) but was lower than those reported in Thailand (62.0%), Mexico (63%) and Mongolia (93.9%) (Abujnah *et al*. 2015; Munkhdelger *et al*. 2017; Ramírez-Castillo *et al*. 2018; Tewawong *et al*. 2020; Yılmaz & Aslantaş 2020). However, the sample size of this study population (60 isolates) was relatively smaller as compared to the mentioned studies, so it may not be able to reflect the true prevalence of MDR in our geographical area.

For the beta-lactamases genes tested, *bla*_TEM_ (36/60; 60.0%) was present prevalently among the UPEC isolates as compared to *bla*_SHV_ (0/60; 0.0%), suggesting that *bla*_TEM_ may be the predominant *bla* genes subtypes in our local scene. Similarly, prior works demonstrated that the extended-spectrum beta-lactamases (ESBL)-producing UPEC isolates harboured a higher prevalence of *bla*_TEM_ but with the absence of *bla*_SHV_ (Alqasim *et al*. 2018; Valadbeigi *et al*. 2020). Recently, *bla*_CTX-M_ has emerged as the leading ESBL gene among the UPEC strains (Alqasim *et al*. 2018). Therefore, the traditional *bla*_SHV_ types may have been replaced by *bla*_CTX-M_, which could explain the absence of *bla*_SHV_ in the current investigation.

In the present study, *tetA* (27/60; 45.0%) was more commonly found among the UPEC isolates as compared to *tetB* (8/60; 13.3%) (Table 4). These results were consistent with earlier studies in Nigeria and Iraq (Olowe *et al*. 2013; Zeadan *et al*. 2022). The predominance of *tetA* may be attributed to the greater transferability of *tetA*, thereby allowing *tetA* to be disseminated more easily among the UPEC strains (Olowe *et al*. 2013).

For the sulphonamide resistance genes, *sul2* (25/60; 41.7%) was more frequently detected among UPEC isolates as compared to *sul1* (13/60; 21.7%) (Table 4). A similar result was reported by Lin *et al*. (2016), where a higher prevalence of *sul2* was observed among the co-trimoxazole-resistant isolates. In addition, a prior study revealed that the minimum inhibitory concentration of co-trimoxazole required to kill the *sul2*-positive bacteria strains was the highest as compared to *sul1* and *sul3-*positive strains, indicating that the drug-resistant activity of the *sul2* was the strongest (Lai *et al*. 2019).

Despite being present prevalently in beta-lactam antimicrobials, including ampicillin, ciprofloxacin and levofloxacin, *bla*_TEM_ was also more frequently detected among non-beta-lactam antimicrobial such as tetracycline in the current investigation (Table 4). Similarly, *sul1* and *sul2* sulphonamide resistance genes were present prevalently among other classes of antimicrobials apart from co-trimoxazole (Table 4). Likewise, *tetA* tetracycline resistance gene was also more frequently detected among the ampicillin, nalidixic acid and co-trimoxazole-resistant isolates (Table 4). These findings agreed with previous studies where positive correlations between the resistance genes and other non-paired antimicrobials were also reported (Jiang *et al*. 2021). These resistance genes are usually located in plasmids or integrons that can be transmitted and acquired easily through numerous horizontal gene transfer events, which may eventually lead to the accumulation of multiple resistance genes (Liu *et al*. 2022).

Although the UPEC strains collected from males and age group 60–79 were significantly resistant to cephalosporin antibiotics (*p* < 0.05) in our previous study (Chin *et al*., 2023), we demonstrated that the patient’s age and gender were not significant risk factors for the carriage of targeted antimicrobial genes in this study. Although some of the resistance genes, such as *tetA* and *bla*_TEM_ were present prevalently among female patients and age group 15–59, their associations were not statistically significant (all *p >* 0.05) (Table 5). This may be due to the insufficient sample size or uneven distribution of UPEC isolates among different age groups and gender, which hinders from detection of the true associations.

## CONCLUSION

In conclusion, we demonstrated the high carriage of *tetA*, *sul1*, *sul2* and *bla*_TEM_ antimicrobial resistance genes among the MDR, ampicillin and tetracycline-resistant isolates. In contrast, *tetB* displayed no significant relationship with any of the antimicrobials tested and *bla*_SHV_ was not detected among the UPEC isolates. The patient’s age and gender were not the risk factors for the carriage of the resistance genes in this study. Comprehensive surveillance programs and close monitoring of resistance genes may be urgently needed to observe the antimicrobial resistance issue in our community.

## Figures and Tables

**Table 1:** Primer sequences and PCR conditions for antimicrobial resistance genes detection.

Reaction	Target gene	Sequence (5′ – 3′)	Size (bp)	Conditions	References
Duplex I	*bla*_TEM_	F: 5′-ATCAGCAATAAACCAGC-3′ R: 5′-CCCCGAAGAACGTTTTC-3′	516	5 min at 94° C, 32 cycles of 30 s at 94° C, annealing for 30 s at 56° C, elongation for 1 min at 72° C, and extension for 10 min at 72° C	Mabilat & Courvalin (1990)
	*bla*_SHV_	F: 5′-AGGATTGACTGCCTTTTTG-3′ R: 5′-ATTTGCTGATTTCGCTCG-3′	392		Colom *et al. (*2003)
Duplex II	*tetA*	F: 5′-GTGAAACCCAACATACCCC-3′ R: 5′-GAAGGCAAGCAGGATGTAG-3′	888	5 min at 94° C, 30 cycles of 1 min at 94° C, annealing for 30 s at 56° C, elongation for 1 min at 72 ° C, and extension for 8 min at 72° C	Maynard *et al*. (2003)
	*tetB*	F: 5′-CCTCAGCTTCTCAACGCGTG-3′ R: 5′-GCACCTTGCTGATGACTCTT-3′	634		Dormanesh *et al. (*2014)
Duplex III	*sul1*	F: 5′-CGGCGTGGGCTACCTGAACG-3′ R: 5′-GCCGATCGCGTGAAGTTCCG-3′	432	5 min at 95° C, 30 cycles of 30 s at 95° C, annealing for 30 s at 58° C, elongation for 45 s at 72° C, and extension for 5 min at 72° C	Shinu *et al. (*2020)
	*sul2*	F: 5′-GCGCTCAAGGCAGATGGCATT-3′ R: 5′-GCGTTTGATACCGGCACCCGT-3′	293		Shinu *et al*. (2020)

**Table 2:** Demographic data of the study population.

Age group	No. of isolates (%)		

	Female (*n =* 44)	Male (*n =* 16)	Total (*n =* 60)
0–14	2 (50.0)	2 (50.0)	4 (6.7)
15–59	20 (80.0)	5 (20.0)	25 (41.7)
≥ 60	22 (71.0)	9 (29.0)	31 (51.7)

**Table 3:** Antimicrobial resistance profiles among the UPEC isolates.

Antimicrobial	No. of resistant isolates (%)
Penicillin	
Ampicillin	43 (71.7)
Tetracyclines	
Tetracycline	31 (51.7)
Minocycline	1 (1.7)
Quinolones	
Nalidixic acid	30 (50.0)
Ciprofloxacin	19 (31.7)
Levofloxacin	16 (26.7)
Sulphonamide	
Co-trimoxazole	20 (33.3)
Phenicol	
Chloramphenicol	10 (16.7)
Carbapenem	
Imipenem	0 (0.0)
Multidrug resistance	24 (40.0)

**Table 4:** Prevalence of resistance genes among different classes of resistant isolates.

Antimicrobial	No. of resistant isolates that conferred the corresponding resistance gene (%)				

	*tetA* (*n =* 27)	*tetB* (*n =* 8)	*sul1* (*n =* 13)	*sul2* (*n =* 25)	*bla*_TEM_ (*n =* 36)
Ampicillin (*n =* 43)	26 (60.5)^a^	7 (16.3)	13 (30.2)^a^	25 (58.1)^a^	30 (69.8)^a^
Tetracycline (*n =* 31)	25 (80.6)^a^	7 (22.6)	12 (38.7)^a^	21 (67.7)^a^	25 (80.6)^a^
Minocycline (*n =* 1)	0 (0.0)	1 (100.0)	0 (0.0)	0 (0.0)	1 (100.0)
Nalidixic acid (*n =* 30)	18 (60.0)^a^	5 (16.7)	10 (33.3)^a^	17 (56.7)^a^	21 (70.0)
Ciprofloxacin (*n =* 19)	12 (63.2)	3 (15.8)	8 (42.1)^a^	11 (57.9)	15 (78.9)^a^
Levofloxacin (*n =* 16)	10 (62.5)	2 (12.5)	8 (50.0)^a^	10 (62.5)^a^	13 (81.3)^a^
Co-trimoxazole (*n =* 20)	15 (75.0)^a^	4 (20.0)	10 (50.0)^a^	16 (80.0)^a^	15 (75.0)
Chloramphenicol (*n =* 10)	5 (50.0)	2 (20.0)	6 (60.0)^a^	5 (50.0)	4 (40.0)
Multidrug resistance (*n =* 24)	19 (79.2)^a^	6 (25.0)	12 (50.0)^a^	16 (66.7)^a^	19 (79.2)^a^

*Notes: bla*_SHV_ was not detected among the UPEC isolates; All the UPEC isolates were susceptible to imipenem.

a indicates *p <* 0.005.

**Table 5:** Prevalence of antimicrobial resistance genes among different genders and age groups.

Resistance gene	No. of isolates (%)							

	Gender			Age group				Total (*n =* 60)

	Female (*n =* 44)	Male (*n =* 16)	*p*	0–14 (*n =* 4)	15–59 (*n =* 25)	≥ 60 (*n =* 31)	*p*	
*tetA*	20 (45.5)	7 (43.8)	0.907	2 (50.0)	13 (52.0)	12 (38.7)	0.597	27 (45.0)
*tetB*	7 (15.9)	1 (6.3)	0.669	0 (0.0)	2 (8.0)	6 (19.4)	0.332	8 (13.3)
*sul1*	7 (15.9)	6 (37.5)	0.088	0 (0.0)	7 (28.0)	6 (19.4)	0.408	13 (21.7)
*sul2*	18 (40.9)	7 (43.8)	0.844	2 (50.0)	11 (44.0)	12 (38.7)	0.869	25 (41.7)
*bla*_TEM_	28 (63.6)	8 (50.0)	0.340	1 (25.0)	16 (64.0)	19 (61.3)	0.328	36 (60.0)

*Note*: *bla*_SHV_ was not detected among the UPEC isolates.
